# Measurement of Cardiothoracic Ratio on Chest X-rays Using Artificial Intelligence—A Systematic Review and Meta-Analysis

**DOI:** 10.3390/jcm13164659

**Published:** 2024-08-08

**Authors:** Jakub Kufel, Łukasz Czogalik, Michał Bielówka, Mikołaj Magiera, Adam Mitręga, Piotr Dudek, Katarzyna Bargieł-Łączek, Magdalena Stencel, Wiktoria Bartnikowska, Sylwia Mielcarska, Sandra Modlińska, Zbigniew Nawrat, Maciej Cebula, Katarzyna Gruszczyńska

**Affiliations:** 1 Department of Radiology and Nuclear Medicine, Faculty of Medical Sciences in Katowice, Medical University of Silesia, Medyków 14, 40-752 Katowice, Poland; 2 Students’ Scientific Association of Computer Analysis and Artificial Intelligence, Department of Radiology and Nuclear Medicine, Medical University of Silesia in Katowice, 40-752 Katowice, Poland; 3 Professor Zbigniew Religa Student Scientific Association, Department of Biophysic, Faculty of Medical Sciences in Zabrze, Medical University of Silesia, Jordana 19, 41-808 Zabrze, Poland; 4 Department of Diagnostic Imaging, Szpital Specjalistyczny im. Sz. Starkiewicza, 41-300 Dąbrowa Górnicza, Poland; 5 Faculty of Medical Sciences in Katowice, Medical University of Silesia, 40-752 Katowice, Poland; 6 Department of Medical and Molecular Biology, Faculty of Medical Sciences, Medical University of Silesia, 41-808 Zabrze, Poland; 7 Foundation of Cardiac Surgery Development, 41-800 Zabrze, Poland; 8 Department of Biophysics, Faculty of Medical Sciences in Zabrze, Medical University of Silesia, Jordana 19, 41-808 Zabrze, Poland; 9 Individual Medical Practice Maciej Cebula, 40-754 Katowice, Poland

**Keywords:** chest X-ray, convolutional neural network, cardiothoracic ratio, CTR, machine learning

## Abstract

**Background:** Chest X-rays (CXRs) are pivotal in clinical diagnostics, particularly in assessing cardiomegaly through the cardiothoracic ratio (CTR). This systematic review and meta-analysis evaluate the efficacy of artificial intelligence (AI) in automating CTR determination to enhance patient care and streamline diagnostic processes. They are concentrated on comparing the performance of AI models in determining the CTR against human assessments, identifying the most effective models for potential clinical implementation. This study was registered with PROSPERO (no. CRD42023437459). No funding was received. **Methods:** A comprehensive search of medical databases was conducted in June 2023. The search strategy adhered to the PICO framework. Inclusion criteria encompassed original articles from the last decade focusing on AI-assisted CTR assessment from standing-position CXRs. Exclusion criteria included systematic reviews, meta-analyses, conference abstracts, paediatric studies, non-original articles, and studies using imaging techniques other than X-rays. After initial screening, 117 articles were reviewed, with 14 studies meeting the final inclusion criteria. Data extraction was performed by three independent investigators, and quality assessment followed PRISMA 2020 guidelines, using tools such as the JBI Checklist, AMSTAR 2, and CASP Diagnostic Study Checklist. Risk of bias was assessed according to the Cochrane Handbook guidelines. **Results:** Fourteen studies, comprising a total of 70,472 CXR images, met the inclusion criteria. Various AI models were evaluated, with differences in dataset characteristics and AI technology used. Common preprocessing techniques included resizing and normalization. The pooled AUC for cardiomegaly detection was 0.959 (95% CI 0.944–0.975). The pooled standardized mean difference for CTR measurement was 0.0353 (95% CI 0.147–0.0760). Significant heterogeneity was found between studies (I^2^ 89.97%, *p* < 0.0001), with no publication bias detected. **Conclusions:** Standardizing methodologies is crucial to avoid interpretational errors and advance AI in medical imaging diagnostics. Uniform reporting standards are essential for the further development of AI in CTR measurement and broader medical imaging applications.

## 1. Introduction

Chest radiographs (X-rays) are widely used in clinical diagnostics. They are a significant part of imaging procedures worldwide due to their numerous advantages—first and foremost, their low cost, high availability, and widespread use in the imaging diagnosis of many lung and heart diseases [1,2,3]. Chest X-rays account for 40% of the 3.6 billion imaging procedures performed annually [4]. One of the important components of a chest X-ray is the cardiothoracic ratio (CTR), which is the ratio of the width of the heart silhouette (transverse cardiac diameter—TCD) to the widest dimension of the chest (transverse thoracic diameter—TTD) [5]. CTR values of 0.42–0.50 are considered normal, and values above 0.5 indicate enlargement of the cardiac silhouette—cardiomegaly, which is considered a pathology [6]. The cut-off value for a CTR may vary depending on the projection and the specific group of patients studied. According to the literature, a CTR should be determined in a posterior–anterior (PA) projection [7,8], which provides the closest representation of the true dimensions of the heart. Nevertheless, some studies report appropriate CTR values in the anteroposterior (AP) position [6], which proves important when diagnosing patients who are lying down and diagnosing young children. There are many causes of cardiomegaly, which always require additional diagnosis due to the high risk of patient deterioration and even death. The development of heart failure results in a 50% survival rate [9], and conditions involving cardiomegaly, such as myocardial infarction, coronary artery disease, cardiomyopathies, or valvular regurgitation lead precisely to heart failure [10]. Artificial intelligence (AI) has emerged as a promising tool for CTR measurement, offering potential improvements in efficiency and consistency. Despite the technical validation of AI methods, there remains a need for clinical evaluation on large datasets to ensure their reliability and effectiveness. Recent studies, such as the one by Saiviroonporn et al., have explored the utility of AI in assisting radiologists by reducing observer variation and operation time. Their findings indicate that, while AI alone may not yet be suitable for automated CTR measurement due to high variation, AI-assisted methods show promise in improving measurement accuracy and speed [11]. In addition to these advances, evaluating AI performance in medical applications requires rigorous and standardized evaluation tools. For example, the Artificial Intelligence Performance Instrument (AIPI) has been developed and tested for its reliability and validity in evaluating AI performance in clinical settings. A study by Lechien et al. evaluated the AIPI using the medical records of otolaryngology patients, comparing AI-generated and physician-generated scores for differential diagnosis, management, and treatment. The AIPI demonstrated adequate internal consistency and moderate-to-strong test–retest reliability, supporting its potential as a robust tool for assessing AI performance in medicine [12]. In this article, we will present a review and meta-analysis of studies using AI to determine the CTR. This study is concentrated on comparing the performance of AI models in determining the CTR against human assessments, identifying the most effective models for potential clinical implementation. The findings from this analysis will assess whether AI will help improve patient care during the diagnosis of cardiomegaly-related diseases, as well as evaluate the potential benefits for radiology and diagnostic imaging specialists by automating the process and speeding up their work.

## 2. Protocol Registration

This study was registered in PROSPERO no. CRD42023437459 [13].

Study protocol supplement: study protocol.

## 3. Materials and Methods

### 3.1. Search Strategy and Selection Criteria

In June 2023, a search of medical databases such as MEDLINE via. PubMed, Scopus, Web of Science, Embase, and Cochrane Library was conducted.

The search strategy was developed in accordance with PICO [14]. A detailed search strategy and PICO schema are presented in (Supplement S1: PICO).

Initially, a total of 173 articles were found in all databases. After applying filters, 117 articles remained. The articles were then entered into the Rayyan tool [15], and 62 duplicates were automatically detected, of which 40 were removed.

Next, 77 articles were analysed by two independent researchers. The analysis looked at titles, abstracts, and keywords. A total of 63 articles were excluded for the following reasons: different form of study from the article (n = 3), wrong study design (n = 35), paediatric population (n = 3), article had no abstract (n = 1), article was published more than 10 years ago (n = 1), and concerning computed tomography (CT) instead of CXRs (n = 19). During the review of articles, one more article was rejected because it used a database of paediatric patients.

Fourteen articles were included in the review (Supplement S2: PRISMAflow diagram). All decisions made by the investigators were recorded—Researcher 1: 62 excluded, 14 included, perhaps 1; Researcher 2: 61 excluded, 14 included, perhaps 2. Conflicts were resolved by a third independent researcher. Inter-investigator concordance was assessed using Cohen’s Kappa test [16], which was 0.87 (percent concordance: 96.15), which is interpreted as near-perfect concordance.

#### Inclusion and Exclusion Criteria

Inclusion criteria: original articles, articles having an abstract, and articles from the last 10 years. Concerning assessment of the cardiothoracic ratio (CTR) on CXRs in the standing position in posterior–anterior (PA) projection using AI.

Exclusion criteria: systematic reviews, meta-analyses, congress abstracts, conference abstracts, paediatric population articles, and other forms of scientific publications that are not original articles. Studies using imaging techniques other than X-rays, e.g., CT, ultrasound, MRI. All studies older than 10 years. Studies that did not use AI. Studies not involving the chest. Studies in which the CTR was not measured using AI.

### 3.2. Data Extraction

All articles meeting the inclusion criteria were subjected to data extraction by three independent investigators. The following information was extracted from each study:

- General characteristics (authors, country, publication date, journal, IF, number of citations, general purpose of article, keywords);
- Dataset (number of examinations, no. of unique patients (if available, male/female), number of CXRs allocated to training, validation, and test with percentage division);
- Characteristics of the AI technology (model’s name, image preprocessing, number of layers, training device);
- Algorithm performance and validation;
- Cardiothoracic ratio (CTR) (average values);
- Results (sensitivity, specificity, AUROC, accuracy, negative predictive value (NPV), positive predictive value (PPV));
- Evidence generation: results (technological, clinical, economic, side effects), comparators/gold standard;
- Ethical, legal, and social considerations.

### 3.3. Quality Assessment

The review article and meta-analysis were conducted in accordance with the PRISMA 2020 Statement (Supplement S3: PRISMA_2020_checklist.docx) and PRISMA 2020 for abstract (Supplement S4: PRISMA_2020_abstract_checklist.docx) [17]. The systematic review was assessed using the JBI Checklist (Supplement S5: JBI_checklist) [18], AMSTAR 2 (Supplement S6: AMSTAR2), an assessment scale for systematic reviews of health interventions [19], and the Critical Appraisal Skills Program, CASP (Supplement S7: CASP checklist), a diagnostic study checklist systematic review [20]. 

### 3.4. Risk of Bias Assessment

All articles were assessed for risk of bias according to the Cochrane Handbook guidelines for randomized and non-randomized controlled trials [21] by two independent reviewers. Discrepancies were resolved by consensus. The Cochrane risk of bias assessment tool covers bias in sample selection, study design, detection of results, data loss, and reporting of results for randomized trials. The assessment is based on three options: low risk, high risk, and unclear risk (Supplement S8: Risk of bias assessment).

### 3.5. Statistical Analysis

The AUC of AI models in cardiomegaly detection was compared between included studies, and the pooled measure was evaluated according to a random effect model due to the high heterogeneity of results (I^2^ > 50%). Additionally, mean difference in CTR measurements between AI models and reference methods were also compared and pooled using the random effect model as standardized mean difference. Q-test and I^2^ values were applied to assess heterogeneity between studies. I^2^ values were interpreted as follows: 25–50%—low heterogeneity, 50–75%—moderate heterogeneity, and above 75%—high heterogeneity. The AUC of each study and pooled AUC was shown in the forest plot. Additionally, publication bias was evaluated using a funnel plot. MedCalc (20.116) was used to perform all statistical analyses. *p* values ≤ 0.05 were considered significant.

### 3.6. Systematic Literature Review Process

The systematic literature review process is presented collectively in (Supplement S9: SLR illustration).

## 4. Results

### 4.1. Aim of This Study

The aim of this study was to compare the performance of CTR determination and segmentation of the heart and lungs by a deep learning model compared to humans [11,22,23,24,25,26,27,28]. This study was conducted to determine the potential of the model to automatically determine the CTR and detect cardiomegaly [29,30,31]. A study by Saivioonporn et al. compared the performance of four deep learning models to determine the most effective one for clinical implementation [32]. A study by Ajmera et al. focused on evaluating the effectiveness of a radiologist in diagnosing cardiomegaly with and without AI [33]. Que et al.’s study focused on demonstrating that a combination of segmentation and deep learning, using the CardioXNet algorithm proposed by the researchers, allows for an automatic diagnosis of heart disease [34]. The characteristics of the studies included in this article are presented in Table 1.

### 4.2. Database

In the 14 studies described by the researchers, the total number of CXR images was 70,742. Only seven studies [11,24,26,28,29,30,32] reported the number of unique patients; thus, it is not possible to determine the total number of unique patients for the entire study. The study with the smallest number of CXR images included 103 of them [34], and the largest included 16,903 [32]. When describing the datasets, the authors of four papers [22,23,28,29] provided complete information on the division of these studies into individual groups (training, validation, test). The authors of 8 papers [22,23,24,28,29,31,32,34] used preexisting datasets, of which the JSRT dataset was used most frequently (6/8 using preexisting datasets).

The full characteristics of the datasets used in this study are shown in Table 2.

### 4.3. Characteristics of the AI Technology

When describing the characteristics of the AI technology, the authors focused on identifying the model name and software, as well as describing the training device and training. Each researcher included information on the model name [11,22,23,24,25,26,27,28,29,30,31,32,33,34]. Only four researchers provided information on the name of the software [11,27,28,29]. Seven researchers did not describe the training device [11,24,25,27,30,33,34]; also, training was not described by seven authors [11,24,25,26,27,28,34].

All characteristics regarding the AI technology used are included in Table 3.

### 4.4. Preprocessing CXRs

More than half of the studies included in this review have used some form of image data preprocessing or image augmentation. Research conducted by Saiviroonporn [11], Lee [29], Chou [30], and Que [34] did not include or did not provide any information on preprocessing methods. Also, Saiviroonporn [32] and Ajmera [33] reported in their papers that only augmentation was used, without any further explanation. Among the preprocessing methods, the most often used were resizing, normalization (grey scale, brightness, and contrast), format conversion, and ROI selection. The augmentation process mostly included cropping, inverting, horizontal flip, contrast scaling, brightness shifting, noise addition, rotation, and scaling.

The complete list of methods used in the included articles is presented in Table 4.

### 4.5. Results

One of the authors described the sensitivity and specificity of cardiomegaly classification [11], while five other researchers described the sensitivity and specificity of CTR calculation [23,24,25,27,34]. Four authors included information on the accuracy of cardiomegaly classification [11,26,28,34], while five researchers described the accuracy of CTR measurement [23,25,27,30,33]. AURC of cardiomegaly classification was included by two investigators [11,34], as was the AUROC of CTR calculation [23,24]. Three investigators described the F1 of cardiomegaly classification [11,28,34], with one describing the F1 of CTR calculation [33]. Four investigators reported the mean error I standard deviation of CTR calculation [23,25,29,33].

The exact results obtained by the researchers are included in Table 5.

### 4.6. Technological, Clinical, and Economic Side Effects

The primary and most significant effect of automating the measurement of CTR mentioned by all authors is the reduction in the duration of this procedure and the increase in accuracy compared to manual measurements. This opens up the possibility to use this test on a routine screening basis. The CTR is a clinically useful indicator for experienced clinicians, so its determination is not intrinsic to every CXR analysis; the gain/loss balance of CTR determination largely depends on the specific clinical situation. The popularization of CTR determination by AI methods will provide a useful diagnostic factor for the CXRs described, without additional financial or time investment.

### 4.7. Comparators/Gold Standard

The studies described in this systematic review unanimously describe manual measurement as the primary and most widely used method for determining the CTR. The most obvious limitation of this method is its time-consuming nature, especially when determining on large datasets. Furthermore, the use of the CTR is highly dependent on individual expertise. A CTR > 0.55 is recognised as cardiomegaly [11]. The first studies using AI to determine the CTR have been conducted between 2018 and 2020, but nowadays, in the clinical setting, a radiologist’s assessment is required to evaluate the compatibility of the measurement with the manual method [11,32,33]. The advantage of AI methods is their time- and labour-saving capabilities [27]. CTR determination methods using AI can be divided into classification-based and segmentation-based methods. Among these, methods using segmentation are more accurate—these are a solution for providing information about the location or extent of the cardiomegaly region, rather than just a binary decision about its presence or absence. These also provide a numerical estimate of the CTR. The downside of this solution is the need to perform segmentation of the X-ray image before starting the network training process [23,35]. The accuracy of the measurement can be further confirmed by performing other high-resolution imaging studies—CMRI and CCT. One study deserves special attention because, in addition to radiologist determination of the CTR on the X-ray, it was confirmed by an ultrasound performed on the same day. The cardiac dimension component was therefore confirmed by another method to increase the reliability of the measurement. However, the AI algorithm can also reflect the inadequacies of an echocardiographic examination when provided with data from it [24].

### 4.8. Meta-Analysis

**Figure 1:**
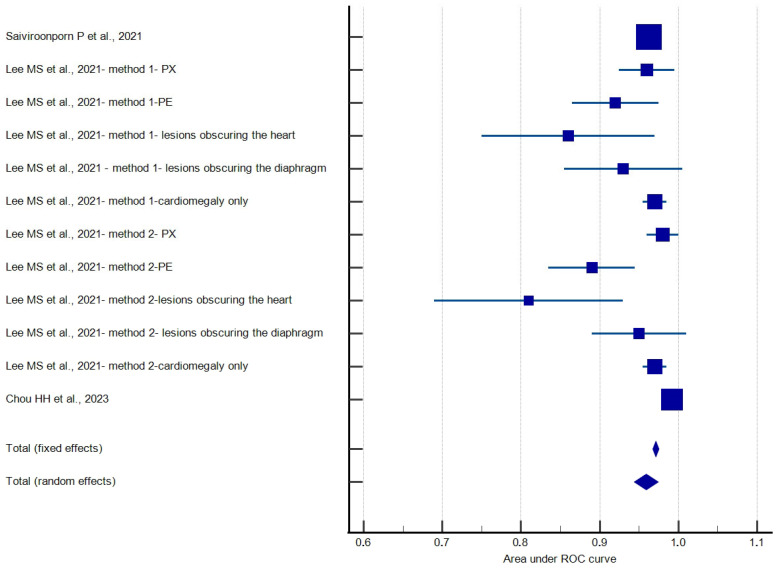
Forest plot of AUC meta-analysis [11,23,29].

**Figure 2:**
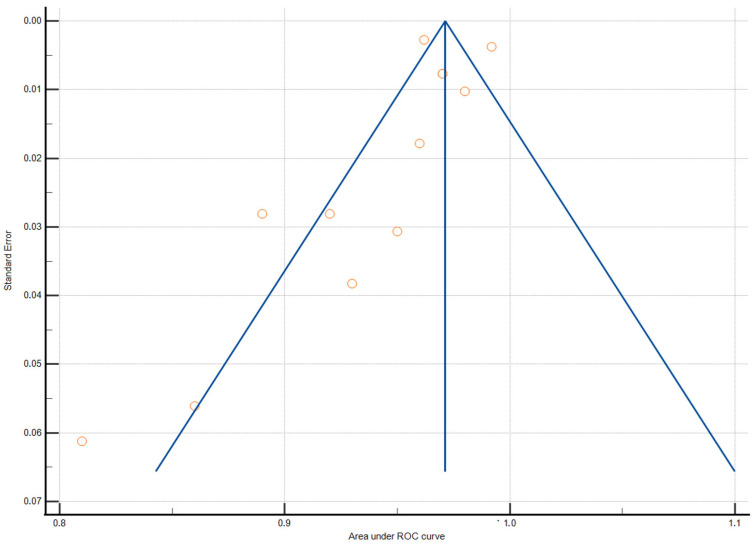
Funnel plot of AUC meta-analysis. Egger’s test (*p* = 0.16), Begg’s test (*p* = 0.07).

The pooled AUC for cardiomegaly detection was 0.959 (95% CI 0.944–0.975, (Table 5). Heterogeneity tests indicated high heterogeneity between studies; it yielded an I^2^ value of 83.76%, indicating significant variability among the studies (Table 6). No publication bias was found, which was evaluated by Egger’s test (*p* = 0.16) and Begg’s test (*p* = 0.07), (Figure 1 and Figure 2).

**Figure 3:**
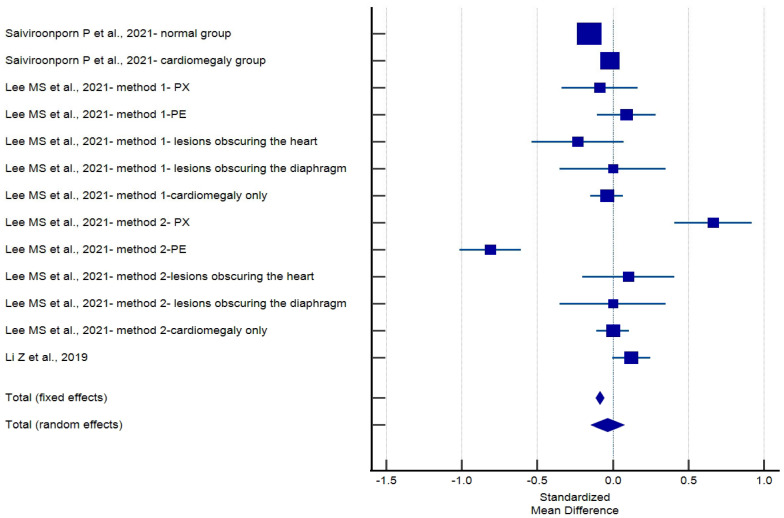
Forest plot of standardized mean difference meta-analysis [11,27,29].

**Figure 4:**
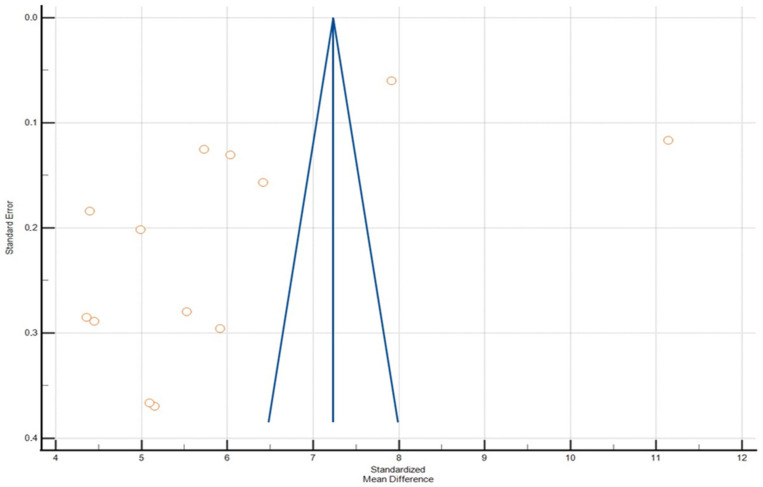
Funnel plot of mean difference meta-analysis using Egger’s test (*p* = 0.43) and Begg’s test (*p* = 0.58).

Absolute value of the pooled standardized mean difference for CTR measurement was 0.0353 (95% CI 0.147–0.0760, Table 7, Figure 3), which is classified as a small effect according to convention by Cohen (2002). Heterogeneity tests revealed substantial variation among studies, with an I² value of 89.97%, indicating significant differences in their findings (Table 8 and Table 9). No publication bias was found, which was evaluated by Egger’s test (*p* = 0.16) and Begg’s test (*p* = 0.07), (Figure 4).

## 5. Discussion

Assessment of the cardiothoracic ratio (CTR) based on chest radiographs is a pivotal diagnostic tool in clinical practice. Often, it sheds initial light on cardiac conditions in patients, providing valuable insights for further diagnostic directions to physicians worldwide. According to Celik et al., early changes in heart failure can be tracked using artificial intelligence through sequential chest radiography over time, potentially playing a significant role in patient monitoring and providing essential data for further research to ultimately determine the application of this method [34,36]. Despite its widespread use and clinical significance, manual CTR measurement remains time-consuming and susceptible to human errors, especially in repeated studies at regular intervals. The advent of artificial intelligence has initiated promising progress in automating CTR determination, offering an alternative to manual measurements.

This systematic review and meta-analysis aimed to evaluate studies employing artificial intelligence for CTR determination and segmentation. The analysis sought to assess whether implementing artificial intelligence could enhance patient care in diagnosing conditions related to cardiomegaly and provide potential benefits to radiologists and imaging specialists by streamlining the diagnostic process.

The studies included in this work underscore the significance of artificial intelligence in automating CTR determination. The findings suggest that artificial intelligence models hold promise for accurately identifying the CTR and diagnosing cardiomegaly, potentially revolutionizing the diagnostic and therapeutic process. Among the analysed studies, the most favourable outcomes were observed in works conducted by Chou et al.. and Lee et al. (method 2—PX), with AI assistance demonstrating high specificity and sensitivity in diagnosing cardiomegaly [23,29].

Integrating artificial intelligence technology with CTR determination brings numerous benefits, particularly in reducing the time required for study evaluation and increasing diagnostic accuracy compared to manual measurements. This automation could facilitate routine screening for heart diseases, providing clinicians with valuable diagnostic information without imposing additional financial and time burdens. Kim et al. suggest that the Emergency Department could be an excellent example of AI utilization for CTR measurements, where a rapidly acting AI model could prioritize patients for cardiac examination based on initial assessments [37].

Furthermore, our analysis highlighted the importance of artificial intelligence-based segmentation methods in enhancing diagnostic accuracy. By providing detailed information on the location and extent of cardiomegaly, segmentation approaches enable numerical CTR assessment, enhancing diagnostic precision. However, it is worth noting that segmentation methods may require the preprocessing of CXR images and additional computational resources.

Additionally, it is possible to further integrate the work of various artificial intelligence models to improve patient care. The integration of Support Vector Machine (SVM) models with AI systems that automatically assess the CTR on frontal chest radiographs can significantly enhance clinical and radiologic management. Initially, an AI model measures CTR, providing accurate detection of cardiac enlargement. The SVM model then classifies patients based on the severity of cardiomegaly, facilitating personalized treatment plans and supporting clinical decisions. This approach not only improves diagnostic precision but also tailors therapeutic strategies, demonstrating the synergistic potential of combining advanced AI technologies in optimizing patient outcomes and clinical workflows [38].

This meta-analysis provides significant insights into the utility of artificial intelligence in CTR measurement, yet certain limitations must be acknowledged. Our limitations on the selection of articles included in the review and meta-analysis have been described above, but we also want to specify other relevant research issues.

Unfortunately, not all studies could be included due to glaring deficiencies in result reporting in some articles, precluding their fair comparison and inclusion in our study. In some publications, we could observe the omission of important data or the lack of a detailed description of the methodology, which could lead to inaccuracies. Another issue, also stemming from the lack of consistent reporting guidelines, was the differences in measurement units used by individual authors, leading to measurement errors.

Therefore, it is so important that scientists conducting research publish detailed data about the study group and precisely describe the steps in the methodology. Creating generalized reporting criteria for researchers, which would include detailed guidance, would be a huge help in this matter. In our opinion, this is a necessary step to standardize future publications. Transparency in reporting, standardized methods, and awareness of potential bias are essential for obtaining credible meta-analytical results.

Implementing uniform criteria in this field will undoubtedly contribute to future research endeavours similar to ours but encompassing a broader range of studies.

Another challenge, also resulting from the lack of coherent reporting guidelines, was the differences in measurement units used by individual authors; hence, in comparing individual data, we opted to use SMD (standardized mean difference), allowing for a comparison of measures used in studies despite discrepancies in applied units.

The Cochrane Q test yielded a statistically significant result (*p* < 0.0001), indicating substantial heterogeneity among the studies included in the meta-analysis. The I^2^ statistic further confirmed a high degree of inconsistency (83.76%) in the results of different studies, suggesting considerable variability beyond what could be expected by chance alone. This high level of heterogeneity may arise from differences in study designs, populations, interventions, or outcome measures among the included studies.

Given the observed significant heterogeneity, it is important to interpret the results of the meta-analysis cautiously. Future studies should aim to identify and address potential sources of heterogeneity to improve the credibility and generalizability of results. Sensitivity analyses or subgroup analyses may be warranted to examine the impact of specific study characteristics on observed heterogeneity and assess the reliability of meta-analytical results. Furthermore, comparing AI-assisted CTR determination with manual measurements highlights the potential for discrepancies and the need for standard validation protocols. Future research efforts should focus on refining algorithms, optimizing preprocessing techniques, and conducting rigorous clinical evaluations to establish the reliability and effectiveness of AI-assisted CTR determination.

In summary, our systematic review and meta-analysis underscore the transformative potential of artificial intelligence in automating CTR determination and enhancing diagnostic accuracy in cardiomegaly. By harnessing artificial intelligence technologies, physicians can streamline the diagnostic process, improve patient care, and mitigate challenges associated with manual measurements. However, further research and clinical validation are essential to ensure the smooth integration of artificial intelligence into clinical practice and maximize its utility in diagnosing cardiovascular diseases and treatment.

## 6. Conclusions

The recent progress in AI within the field of medicine has been impressive, garnering increasing attention from researchers. This paper highlights the promising role of artificial intelligence in measuring the cardiothoracic ratio (CTR).

Our findings indicate that the application of AI in assessing the CTR on chest X-rays (CXRs) can significantly shorten the diagnosis time for patients and could be integrated routinely into imaging studies. This integration is likely to alleviate the workload of radiologists, improving efficiency and accuracy in clinical settings. Furthermore, AI methods have shown potential in reducing observer variation and operation time, aligning with the goals of improving consistency and reliability in CTR measurement. However, further research is needed, particularly based on standardized and coherent methodologies. Inconsistencies in reporting methods and differences in measurement units between studies may lead to interpretational errors, hindering the advancement of AI in this direction due to the inability to leverage existing results and compare them. Adopting uniform reporting standards and methodologies is crucial and indispensable for future research in this field, facilitating and accelerating the further development of AI methods not only in determining CTR but also in all aspects of medical imaging diagnostics.

## Figures and Tables

**Table 1:** Characteristics of type of study.

Study No.	Authors	Type of Study (Classification, Segmentation, Both)
1	Saiviroonporn P et al. [11]	classification
2	Saiviroonporn P et al. [32]	classification
3	Arsalan M et al. [22]	both
4	Lee MS et al. [29]	both
5	Chou CY et al. [30]	classification
6	Chou HH et al. [23]	both
7	Nam JG et al. [24]	both
8	Ajmera P et al. [33]	both
9	Que Q et al. [34]	both
10	Jafar A et al. [31]	both
11	Chaisangmongkon W et al. [25]	both
12	Zhou L et al. [26]	both
13	Li Z et al. [27]	both
14	Wu JX et al. [28]	both

**Table 2:** Characteristics of included studies—databases.

Study No.	Authors	No. of Examinations		Training	Validation	Test	Preexisting Dataset Name
		No. of Unique Patients	No. of CXRs	No. of CXRs	No. of CXRs	No. of CXRs	
1	Saiviroonporn P et al. [11]	7352	7352	-	-	-	-
2	Saiviroonporn P et al. [32]	16,903	16,903	-	7517	9386	JSRT dataset, Montgomery County dataset, ChestX-ray14 dataset, CheXpert dataset
3	Arsalan M et al. [22]	-	4919	4331	20	568	JSRT dataset, Montgomery County dataset, Shenzhen X-Ray
4	Lee MS et al. [29]	1793	1793	634	159	1000	JSRT dataset
5	Chou CY et al. [30]	2474	12,391	-	-	537	-
6	Chou HH et al. [23]	-	927	460	54	413	JSRT dataset
7	Nam JG et al. [24]	5277	5384	-	107	-	-
8	Ajmera P et al. [33]	-	3599	3416	-	183	-
9	Que Q et al. [34]	-	103	-		-	ChestX-ray8 Database
10	Jafar A et al. [31]	-	247	-	-	-	JSRT dataset
11	Chaisangmongkon W et al. [25]	-	8316	-	-	-	JSRT dataset, The Montgomery County dataset, ChestX-ray14 dataset, CheXpert dataset, Proprietary dataset
12	Zhou L et al. [26]	2638	2838	2554	284	-	-
13	Li Z et al. [27]	5000	5500	4000	1000	500	-
14	Wu JX et al. [28]	-	200	-	-	-	NIH CXR dataset

**Table 3:** Characteristics of AI technology.

Study No.	Authors	Models Name	Software	Training Device	Training
1	Saiviroonporn P et al. [11]	VGG-16 U-Net	MATLAB software (R2019a, MathWorks, Inc., Natick, MA, USA)	-	-
2	Saiviroonporn P et al. [32]	AlbuNet, SegNet, VGG-11, and VGG-16	-	Nvidia Tesla V100 GPU 32 GB	ImageNet
3	Arsalan M et al. [22]	X-RayNet-1 and X-RayNet-2	-	Intel Core™ i7-3770K CPU with the clock speed of 3.50 GHz (4 cores). The system RAM was 28 GB with NVIDIA GeForce GTX Titan X GPU (3072 Cuda cores with a graphics memory of 12 GB)	MATLAB 2019a
4	Lee MS et al. [29]	U-Net, XLSor	PyTorch framework (2.4)	GTX Titan XP; NVIDIA, Santa Clara, CA, USA	ImageNet pre-trained ResNet-101
5	Chou CY et al. [30]	U-Net	-	-	iCTR Assessment System
6	Chou HH et al. [23]	AlbuNet-34, ResNet34	-	NVIDIA RTX 3090	ImageNet
7	Nam JG et al. [24]	DensNet, ResNet152	-	-	-
8	Ajmera P et al. [33]	Attention U-Net DL	-	-	ImageNet
9	Que Q et al. [34]	U-Net, Dense-Net, CardioXNet	-	-	-
10	Jafar A et al. [31]	CardioNet-B	-	Intel^®®^ Core™ i7-8700 CPU	MATLAB R2021
11	Chaisangmongkon W et al. [25]	VGG-11 U-Net, VGG-16 U-Net, SegNet i AlbuNet	-	-	-
12	Zhou L et al. [26]	U-Net	-	Intel Xeon Processor E5–2620 v4 2.10 GHz central processing unit and four 12GB memory NVIDIA GeForce GTX 1080 Ti	-
13	Li Z et al. [27]	U-Net	ITK-SNAP (4.2.0)	-	-
14	Wu JX et al. [28]	DenseNet, ResNet, FC-ResNets, UNet	MATLAB software, LabVIEW (NITM, Austin, TX, USA)	NVIDIA ^®^ GeForce ^®^ RTX™ 2080 Ti, 1755 MHz, 11 GB GDDR6	-

**Table 4:** Preprocessing and augmentation.

Study No.	Authors	Preprocessing	Augmentation
1	Saiviroonporn P et al. [11]	No data	No data
2	Saiviroonporn P et al. [32]	No data	Expanded augmentation repertoire
3	Arsalan M et al. [22]	Not required	Cropping, resizing, and horizontal flipping with interpolation
4	Lee MS et al. [29]	No data	Not applied
5	Chou CY et al. [30]	No data	No data
6	Chou HH et al. [23]	resizing to 512 × 512 pxl	RandomResizedCrop, ShiftScaleRotate, RandomBrightnessContrast, InverImg, ElasticTransform, GridDistortion, and OpticalDistorsion
7	Nam JG et al. [24]	resizing to 224 × 224 or 299 × 299 pxl	Inversion, Fast Autoaugment
8	Ajmera P et al. [33]	No data	Image augmentation
9	Que Q et al. [34]	No data	No data
10	Jafar A et al. [31]	Resizing to 350 × 350 pxl	Cropping, horizontal flipping, translation, and horizontal flipping with translation
11	Chaisangmongkon W et al. [25]	Transformation into 8-bit grayscale PNG images, resizing to 512 × 512 pxl, histogram equalization to normalize brightness and contrast	Augmentation process where each image has a 60% chance of becoming transformed: arithmetic change (contrast normalization), contrast adjustments (gamma and linear contrast scaling), brightness shift, geometric alterations (elastic, perspective, and affine transformations), noise addition (Gaussian blur and additive Gaussian noise), and horizontal flipping
12	Zhou L et al. [26]	Pixel intensity converted into [0, 4095], linear intensity range compressed to [0, 255]	(Conducted on training dataset) Randomized image flipping, contrast and brightness adjusting, rotation with angles between −15° and 15°, optimalisation performed by stochastic gradient descent optimizer
13	Li Z et al. [27]	Normalisation by grey-scale transformation, cropping, resizing to 800 × 800 pxl	Not considered
14	Wu JX et al. [28]	Conversion to TIF, resizing to 420 × 420 pxl, ROI selection	No data

**Table 5:** Results.

Study No.	Authors	Sensitivity	Specificity	Accuracy	AUROC	F1	Mean Error of AI	Standard Deviation of AI	Mean Error of Manual Measurement	SD of Manual Measurement
1	Saiviroonporn P et al. [11]	97.5% ^1^	82.8% ^1^	87.9% ^1^	0.962 ^1^	0.849 ^1^	-	-	-	-
2	Saiviroonporn P et al. [32]	-	-	-	-	-	-	-	-	-
3	Arsalan M et al. [22]	-	-	-	-	-	-	-	-	-
4	Lee MS et al. [29]	-	-	94.97% ^2^	-	-	-	-	-	-
5	Chou CY et al. [30]	-	-	94.97% ^2^	-	-	-	-	-	-
6	Chou HH et al. [23]	96.4% ^2^	91% ^2^	94.9% ^2^	0.992 ^2^	-	0.83% ^2^	1.52% ^2^	1.46% ^2^	-
7	Nam JG et al. [24]	69.6% ^2^	65.8% ^2^	-	0.706 ^2^	-	-	-	-	-
8	Ajmera P et al. [33]	80% ^2^	99% ^2^	94.96% ^2^	-	0.88^2^	0.0254 ^2^	0.006 ^2^	-	-
9	Que Q et al. [34]	-	-	93.75% ^1^	0.93481	0.9434 ^1^				
10	Jafar A et al. [31]	-	-	-	-	-	-	-	-	-
11	Chaisangmongkon W et al. [25]	94.21% ^2^	98.02% ^2^	96.32% ^2^			0.002 ^2^	0.0157 ^2^	−0.0076 ^2^	0.0136 ^2^
12	Zhou L et al. [26]	-	-	98% ^1^	-	-	-	-	-	-
13	Li Z et al. [27]	97.2% ^2^	92.7% ^2^	95.3% ^2^	-	-	0.0004 ^3^	0.0133 ^3^	−0.0083 ^3^	0.00187 ^3^
14	Wu JX et al. [28]	-	-	98.4% ^1^	-	0.9838 ^1^	-	-	-	-

^1^ Cardiomegaly classification; ^2^ CTR measurement. ^3^ CTR measurement between deep learning and reference standard.

**Table 6:** Results of AUC meta-analysis.

Study	ROC Area	Standard Error	95% CI	z	P	Weight (%)	
						Fixed	Random
Saiviroonporn P et al. [11]	0.962	0.00277	0.957 to 0.967	-	-	51.18	15.26
Lee MS et al.—method 1—PX [29]	0.960	0.0179	0.925 to 0.995	-	-	1.23	8.63
Lee MS et al.—method 1—PE [29]	0.920	0.0281	0.865 to 0.975	-	-	0.5	5.22
Lee MS et al.—method 1—lesions obscuring the heart [29]	0.860	0.0561	0.750 to 0.970	-	-	0.12	1.74
Lee MS et al.—method 1—lesions obscuring the diaphragm [29]	0.930	0.0383	0.855 to 1.00	-	-	0.27	3.32
Lee MS et al.—method 1-cardiomegaly only [29]	0.970	0.00765	0.955 to 0.985	-	-	6.71	13.56
Lee MS et al.—method 2—PX [29]	0.980	0.0102	0.960 to 1.000	-	-	3.78	12.33
Lee MS et al.—method 2—PE [29]	0.890	0.0281	0.835 to 0.945	-	-	0.50	5.22
Lee MS et al.—method 2—lesions obscuring the heart [29]	0.810	0.0612	0.690 to 0.930	-	-	0.10	1.49
Lee MS et al.—method 2—lesions obscuring the diaphragm [29]	0.950	0.0306	0.890 to 1.000	-	-	0.42	4.64
Lee MS et al.—method 2—cardiomegaly only [29]	0.970	0.00765	0.955 to 0.985	-	-	6.71	13.56
Chou HH et al. [30]	0.992	0.00372	0.985 to 0.999	-	-	28.47	15.03
Total (fixed effects)	0.971	0.00198	0.967 to 0.975	489.843	<0.001	100.00	100.00
Total (random effects)	0.959	0.00787	0.944 to 0.975	121.924	<0.001	100.00	100.00

**Table 7:** Test of heterogeneity; AUC meta-analysis.

Q	DF	Significance Level	I^2^ (Inconsistency)	95% CI for I^2^
67.7322	11	*p* < 0.0001	83.76%	73.09 to 90.20

**Table 8:** Results of meta-analysis comparing mean difference in CTR measurement between AI models and reference methods. SMD—standardized mean difference, SE—standard error.

Study	N1	N2	Total	SMD	SE	95% CI	t	*p*	Weight %)	
									Fixed	Random
Saiviroonporn P et al.—normal group [11]	4993	4993	9866	−0.157	0.0202	−0.196 to −0.117			48.85	10.29
Saiviroonporn P et al.—cardiomegaly group [11]	2419	2419	4838	−0.0213	0.0288	−0.0776 to 0.0351			24.03	10.16
Lee MS et al.—method 1—PX [29]	123	123	246	−0.0873	0.127	−0.338 to 0.163			1.23	6.85
Lee MS et al.—method 1—PE [29]	202	202	404	0.0911	0.0994	−0.104 to 0.286			2.01	7.91
Lee MS et al.—method 1—lesions obscuring the heart [29]	83	83	166	−0.233	0.155	−0.539 to 0.0730			0.83	5.87
Lee MS et al.—method 1—lesions obscuring the diaphragm [29]	63	63	126	0.000	0.177	−0.351 to 0.351			0.63	5.18
Lee MS et al.—method 1—cardiomegaly only [29]	652	652	1304	−0.0400	0.0554	−0.149 to 0.0686			6.48	9.49
Lee MS et al.—method 2—PX [29]	123	123	246	0.663	0.131	0.406 to 0.921			1.16	6.72
Lee MS et al.—method 2—PE [29]	202	202	404	−0.811	0.103	−1.014 to −0.608			1.86	7.75
Lee MS et al.—method 2—lesions obscuring the heart [29]	83	83	166	0.104	0.155	−0.201 to 0.410			0.83	5.88
Lee MS et al.—method 2—lesions obscuring the diaphragm [29]	63	63	126	0.000	0.177	−0.351 to 0.351			0.63	5.18
Lee MS et al.—method 2—cardiomegaly only [29]	652	652	1304	0.000	0.0554	−0.109 to 0.109			6.48	9.49
Li Z et al. [27]	500	500	1000	0.123	0.0633	−0.000914 to 0.247			4.98	9.23
Total (fixed effects)	10098	10098	20196	−0.0858	0.0141	−0.113 to −0.0582	−6.087	<0.001	100.00	100.00
Total (random effects)	10098	10098	20196	−0.0353	0.0568	−0.147 to 0.0760	−0.622	0.534	100.00	100.00

**Table 9:** Test of heterogeneity; mean difference meta-analysis.

Q	DF	Significance Level	I^2^ (Inconsistency)	95% CI for I^2^
119.6263	12	*p* < 0.0001	89.97%	84.70 to 93.42

## Data Availability

The original contributions presented in this study are included in the article/Supplementary Materials; further inquiries can be directed to the corresponding author.

## Supplementary Materials

The following supporting information can be downloaded at https://www.mdpi.com/article/10.3390/jcm13164659/s1, Supplement: S0–S9, Supplement: Risk of bias assessment [39,40].
