# Supplementary material for: Measurement of Cardiothoracic Ratio on Chest X-rays Using Artificial Intelligence—A Systematic Review and Meta-Analysis
Source: J Clin Med. 2024 Aug 8;13(16):4659. doi: 10.3390/jcm13164659 (PMC11355006; doi:10.3390/jcm13164659)
Supplement: Supplementary file 1 [file jcm-13-04659-s001.zip › Supplement S0_Study protocol.pdf]

# Study protocol

**Article title:** *Measurement of cardiothoracic ratio on chest X-rays using artificial intelligence — a systematic review and meta-analysis*

**Objective:**
The main objective was to conduct a systematic review and meta-analysis to evaluate the effectiveness of artificial intelligence (AI) in determining and segmenting the Cardiothoracic Ratio (CTR) from chest radiographs (X-rays) presented in original research.

**Methods:**

1. **Search Strategy and Selection Criteria**

Comprehensive research was done using different databases such as MEDLINE via. PubMed, Scopus, Web of Science, Embase and Cochrane Library. The search strategy was based on the PICO scheme, thanks to which the entire search strategy was developed and the search terms were selected: Terms:

chest x-ray, radiography, chest roentgenography, thoracic x-ray, cardiothoracic ratio, CTR, artificial intelligence, machine learning, deep learning, neural network

2. **Inclusion and exclusion criteria**

Inclusion criteria for articles used in the study were: original articles, having an abstract, from the last 10 years. Concerning assessment of cardiothoracic ratio (CTR) on CXR in the standing position in posterior-anterior (PA) projection using AI.

In order to obtain the largest possible number of studies meeting the remaining criteria, it was decided to include both prospective and retrospective studies in this meta-analysis.

Exclusion criteria for articles used in the study were: systematic reviews, meta-analyses, congress abstracts, conference abstracts, pediatric population articles, and other forms of scientific publications that are not original articles. Studies using imaging techniques other than X-ray. e.g. CT, ultrasound, MRI. All studies older than 10 years. Studies that did not use AI. Studies not involving the chest. Studies in which CTR was not measured using AI.

3. **Study selection**

The process of selection of the interest studies was conducted by two reviewers. After removing duplicates, they independently screened abstracts to select potential eligible studies. Then, full text reports were analyzed for eligibility. A third reviewer resolved possible discrepancies highlighted during the selection process, if consensus was not found.

4. **Data Extraction**

The following data were extracted by three analysts from the selected articles: According to the common criteria highlighted during the phase 1 of the data extraction, the following items were extracted:

- General characteristics (Authors, country, publication date, journal, IF, number of citation, general purpose of article, keywords).
- Dataset (Number of examinations, no. of unique patients (if available male/female), number of CXR allocated to training, validation and test with percentage division).
- Characteristics of the AI technology (Models name, Image Preprocessing, Number of layers, Training device).
- Algorithm performance and validation.
- Cardiothoracic ratio (CTR) (average values).
- Results (sensitivity, specificity, AUROC, Accuracy, negative predictive value (NPV), positive predictive value (PPV))

- Evidence generation: Results (Technological, clinical, economic, side effects), Comparators / gold standard.
- Ethical, legal, and social considerations.

5. **Quality Assessment**
    a. MI-CLAIM
6. **Checklists**
    a. PRISMA 2020 checklist
    b. PRISMA 2020 abstract checklist
    c. JBI Checklist
    d. AMSTAR 2
    e. MIDQS-AI-SRM: checklist
    f. PICO
7. **Risk of bias assessment**
    a. Cochrane Handbook guidelines for randomised and non-randomised controlled trials
