# Supplementary material for: Measurement of Cardiothoracic Ratio on Chest X-rays Using Artificial Intelligence—A Systematic Review and Meta-Analysis
Source: J Clin Med. 2024 Aug 8;13(16):4659. doi: 10.3390/jcm13164659 (PMC11355006; doi:10.3390/jcm13164659)
Supplement: Supplementary file 1 [file jcm-13-04659-s001.zip › Supplement S1_PICO.pdf]

PICO scheme of searching

PICO elements	Keywords	Search terms	Search strategy
**P** (**P**atient or/and **P**opulation)	Adult patients with a standing chest x-ray in posterior-anterior (PA) projection.	Chest x-ray	Chest X-Ray<br>OR<br>Chest Xray<br>OR<br>Chest X Ray<br>OR<br>Chest Roentgenography<br>OR<br>Radiography, Thoracic<br>OR<br>Thorax X-Ray<br>OR<br>Thorax Xray<br>OR<br>Thorax X Ray
**I** (**I**ntervention)	Measuring the cardiothoracic ratio on a chest x-ray.	Cardiothoracic ratio	Cardiothoracic ratio<br>OR<br>CTR
**C** (**C**omparison)	Patients without a chest X-ray.		
**O** (**O**utcome)	The result of measuring the cardiothoracic ratio on a chest X-ray using artificial intelligence.	Artificial intelligence	Artificial intelligence<br>OR<br>AI<br>OR<br>Machine Learning<br>OR<br>Deep Learning<br>OR<br>Neural Network<br>OR<br>Computer Neural Network

Search strategy (13.06.2023)

String:

(Chest X-Ray OR Chest Xray OR Chest X Ray OR Chest Roentgenography OR Radiography, Thoracic OR Thorax X-Ray OR Thorax Xray OR Thorax X Ray) AND (Cardiothoracic ratio OR CTR) AND (Artificial intelligence OR AI OR Machine Learning OR Deep Learning OR Neural Network OR Computer Neural Network)

PubMed:

(Chest X-Ray OR Chest Xray OR Chest X Ray OR Chest Roentgenography OR Radiography, Thoracic OR Thorax X-Ray OR Thorax Xray OR Thorax X Ray) AND (Cardiothoracic ratio OR CTR) AND (Artificial intelligence OR AI OR Machine Learning OR Deep Learning OR Neural Network OR Computer Neural Network)

28 -> Filters: Abstract, in the last 10 years -> 25

25:
https://pubmed.ncbi.nlm.nih.gov/?term=%28Chest+X-Ray+OR+Chest+Xray+OR+Chest+X+Ray+OR+Chest+Roentgenography+OR+Radiography%2C+Thoracic+OR+Thorax+X-Ray+OR+Thorax+Xray+OR+Thorax+X+Ray%29+AND+%28Cardiothoracic+ratio+OR+CTR%29+AND+%28Artificial+intelligence+OR+AI+OR+Machine+Learning+OR+Deep+Learning+OR+Neural+Network+OR+Computer+Neural+Network%29&filter=simsearch1.fha&filter=datesearch.y_10&sort=relevance

Scopus:
(ALL (chest AND x-ray OR chest AND xray OR chest AND x AND ray OR chest AND roentgenography OR radiography, AND thoracic OR thorax AND x-ray OR thorax AND xray OR thorax AND x AND ray) AND ALL (cardiothoracic AND ratio OR ctr) AND ALL (artificial AND intelligence OR ai OR machine AND learning OR deep AND learning OR neural AND network OR computer AND neural AND network)) AND (LIMIT-TO (PUBYEAR , 2023) OR LIMIT-TO (PUBYEAR , 2022) OR LIMIT-TO (PUBYEAR , 2021) OR LIMIT-TO (PUBYEAR , 2020) OR LIMIT-TO (PUBYEAR , 2019) OR LIMIT-TO (PUBYEAR , 2018) OR LIMIT-TO (PUBYEAR , 2017) OR LIMIT-TO (PUBYEAR , 2016) OR LIMIT-TO (PUBYEAR , 2014) OR LIMIT-TO (PUBYEAR , 2013)) AND (LIMIT-TO (DOCTYPE , "ar"))

69 -> Filters: in the last 10 years, article -> 42

42:
https://www.scopus.com/results/results.uri?sort=cp-f&src=s&nlo=&nlr=&nls=&sid=704527290d94d2d74d004937b9a51e35&sot=a&sdt=cl&sl=193&s=%28ALL%28chest+AND+x-ray+OR+chest+AND+xray+OR+chest+AND+x+AND+ray+OR+chest+AND+roentgenography+OR+radiography%2C+AND+thoracic+OR+thorax+AND+x-ray+OR+thorax+AND+xray+OR+thorax+AND+x+AND+ray%29+AND+ALL%28cardiothoracic+AND+ratio+OR+ctr%29+AND+ALL%28artificial+AND+intelligence+OR+ai+OR+machine+AND+learning+OR+deep+AND+learning+OR+neural+AND+network+OR+computer+AND+neural+AND+network%29%29&origin=resultslist&zone=leftSideBar&editSaveSearch=&txGid=bebdf873af9b5a3af9bb31bc0ea73ad4&sessionSearchId=704527290d94d2d74d004937b9a51e35&limit=10&cluster=scopubyr%2C%222015%22%2Ct%2C%222017%22%2Ct%2C%222018%22%2Ct%2C%222019%22%2Ct%2C%222020%22%2Ct%2C%222021%22%2Ct%2C%222022%22%2Ct%2C%222023%22%2Ct%2Bscosubtype%2C%22ar%22%2Ct

Web of Science:
(Chest X-Ray OR Chest Xray OR Chest X Ray OR Chest Roentgenography OR Radiography, Thoracic OR Thorax X-Ray OR Thorax Xray OR Thorax X Ray) AND (Cardiothoracic ratio OR CTR) AND (Artificial intelligence OR AI OR Machine Learning OR Deep Learning OR Neural Network OR Computer Neural Network) (Topic)

https://www.webofscience.com/wos/woscc/summary/4ea20e05-2b1f-4314-98d4-dfa9d11eb2ea-9159f93e/relevance/1

24 -> Filters: in the last 10 years, article -> 17

Embase:
('chest x-ray'/exp OR 'chest x-ray' OR (('chest'/exp OR chest) AND ('x ray'/exp OR 'x ray')) OR 'chest xray'/exp OR 'chest xray' OR (('chest'/exp OR chest) AND xray) OR 'chest x ray'/exp OR 'chest x ray' OR (('chest'/exp OR chest) AND x AND ray) OR 'chest roentgenography'/exp OR 'chest roentgenography' OR (('chest'/exp OR chest) AND ('roentgenography'/exp OR roentgenography)) OR 'radiography, thoracic'/exp OR 'radiography, thoracic' OR (('radiography,'/exp OR radiography,) AND thoracic) OR 'thorax x-ray'/exp OR 'thorax x-ray' OR (('thorax'/exp OR thorax) AND ('x ray'/exp OR 'x ray')) OR 'thorax xray' OR (('thorax'/exp OR thorax) AND xray) OR 'thorax x ray'/exp OR 'thorax x ray' OR (('thorax'/exp OR thorax) AND x AND ray)) AND ('cardiothoracic ratio'/exp OR 'cardiothoracic ratio' OR (cardiothoracic AND ('ratio'/exp OR ratio)) OR ctr) AND ('artificial intelligence'/exp OR 'artificial intelligence' OR (artificial AND ('intelligence'/exp OR intelligence)) OR ai OR 'machine learning'/exp OR 'machine learning' OR (('machine'/exp OR machine) AND ('learning'/exp OR learning)) OR 'deep learning'/exp OR 'deep learning' OR (deep AND ('learning'/exp OR

learning)) OR 'neural network'/exp OR 'neural network' OR (neural AND ('network'/exp OR network)) OR 'computer neural network'/exp OR 'computer neural network' OR (('computer'/exp OR computer) AND neural AND ('network'/exp OR network)))

51 -> Filters: in the last 10 years, article -> 32

Cochrane Library:
(Chest X-Ray OR Chest Xray OR Chest X Ray OR Chest Roentgenography OR Radiography, Thoracic OR Thorax X-Ray OR Thorax Xray OR Thorax X Ray) AND (Cardiothoracic ratio OR CTR) AND (Artificial intelligence OR AI OR Machine Learning OR Deep Learning OR Neural Network OR Computer Neural Network) in Title Abstract Keyword - (Word variations have been searched)

1 -> Filters: in the last 10 years, article -> 1

Results of search summary:
All articles in scanning (Rayyan) before automated duplicates detect: 117
Duplicates automatically detected: 62
Duplicates deleted: 40
First scanning (Rayyan): 77
The conflict between researchers: 2
Researcher 1: excluded 62, included 14, maybe 1
Researcher 2: excluded 61, included 14, maybe 2
Articles accepted: 14
Articles rejected: 63

Results
% of agreement: 84.35754189944134%

Cohen’s k: 0.564476885644769

Moderate agreement
