# Supplementary material for: Measurement of Cardiothoracic Ratio on Chest X-rays Using Artificial Intelligence—A Systematic Review and Meta-Analysis
Source: J Clin Med. 2024 Aug 8;13(16):4659. doi: 10.3390/jcm13164659 (PMC11355006; doi:10.3390/jcm13164659)
Supplement: Supplementary file 1 [file jcm-13-04659-s001.zip › Supplement S9_ SLR illustration.pdf]

Here is a PICO scheme created by our researchers consisting of Keywords, Search terms and a Search strategy used in our Systematic Review.

**PICO elements**	**Keywords**	**Search terms**	**Search strategy**
**P** (**P**atient or/and **P**opulation)	Adult patients with a standing chest x-ray in posterior-anterior (PA) projection.	Chest x-ray	Chest X-Ray<br>OR<br>Chest Xray<br>OR<br>Chest X Ray<br>OR<br>Chest Roentgenography<br>OR<br>Radiography, Thoracic<br>OR<br>Thorax X-Ray<br>OR<br>Thorax Xray<br>OR<br>Thorax X Ray
**I** (**I**ntervention)	Measuring the cardiothoracic ratio on a chest x-ray.	Cardiothoracic ratio	Cardiothoracic ratio<br>OR<br>CTR
**C** (**C**omparison)	Patients without a chest X-ray.		
**O** (**O**utcome)	The result of measuring the cardiothoracic ratio on a chest X-ray using artificial intelligence.	Artificial intelligence	Artificial intelligence<br>OR<br>AI<br>OR<br>Machine Learning<br>OR<br>Deep Learning<br>OR<br>Neural Network<br>OR<br>Computer Neural Network

We chose and searched databases for articles. Afterwards, using Rayyan, we removed duplicates, screened abstracts and after exclusion of incompatible reports we included 14 articles to our review.
The full Search strategy including exact strings used in individual databases are included in Supplement 1:PICO

The conduction of screening process looked as follows:

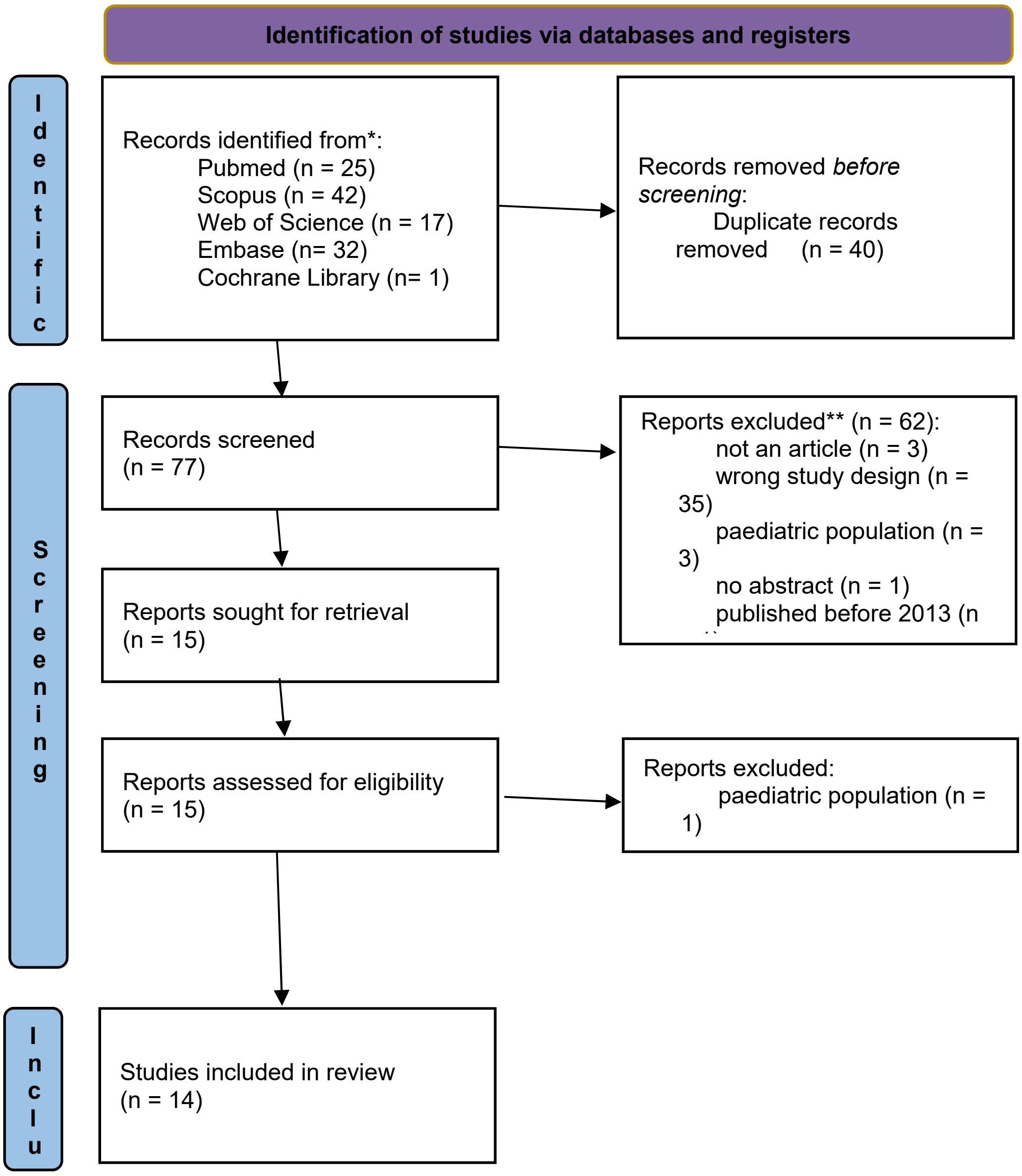

*Consider, if feasible to do so, reporting the number of records identified from each database or register searched (rather than the total number across all databases/registers).

**If automation tools were used, indicate how many records were excluded by a human and how many were excluded by automation tools.

From the included studies we extracted data which is available in Table 4. Then we synthesized the results and derived the conclusions.
